# Junctophilins: Key Membrane Tethers in Muscles and Neurons

**DOI:** 10.3389/fnmol.2021.709390

**Published:** 2021-07-08

**Authors:** Christopher A. Piggott, Yishi Jin

**Affiliations:** Neurobiology Section, Division of Biological Sciences, University of California, San Diego, San Diego, CA, United States

**Keywords:** membrane contact site proteins, ER–PM tethers, calcium channels, RyR channels, jph-1, synaptic transmission, muscle excitation

## Abstract

Contacts between the endoplasmic reticulum (ER) and plasma membrane (PM) contain specialized tethering proteins that bind both ER and PM membranes. In excitable cells, ER–PM contacts play an important role in calcium signaling and transferring lipids. Junctophilins are a conserved family of ER–PM tethering proteins. They are predominantly expressed in muscles and neurons and known to simultaneously bind both ER- and PM-localized ion channels. Since their discovery two decades ago, functional studies using junctophilin-deficient animals have provided a deep understanding of their roles in muscles and neurons, including excitation-contraction coupling, store-operated calcium entry (SOCE), and afterhyperpolarization (AHP). In this review, we highlight key findings from mouse, fly, and worm that support evolutionary conservation of junctophilins.

## Introduction

Close contact sites between membrane compartments are observed universally in all cell types (Prinz et al., 2020). Junctional Membrane Complexes (JMCs) refer to stable contacts between the endoplasmic reticulum (ER) and plasma membrane (PM) found in excitable cells, particularly muscles and neurons. JMCs are composed of unique proteins that couple PM electrical excitation to ER calcium release. This review focuses on the junctophilin protein family, originally identified from rabbit muscle JMCs. Extensive work has now established that junctophilins are the primary component responsible for the generation of JMCs in skeletal and cardiac muscles. Junctophilins directly bind PM- and ER-localized calcium channels, and enable efficient trans-membrane signaling. Here, we will begin with a historical overview on the discovery of junctophilins, then cover functional studies of mammalian junctophilins in muscles and neurons, and end with emerging evidence supporting evolutionary conservation of junctophilins.

### Discovery of Junctophilins at Triad Junctions in Muscle

Muscles have specialized structures known as transverse tubules (t-tubules) that are tubular invaginations of the PM. T-tubules extend the PM deep into the muscle cell where they make JMCs with the sarcoplasmic reticulum (SR, muscle equivalent of ER). In skeletal muscle, t-tubules are sandwiched between two SR compartments. Under electron microscopy, the t-tubule and adjacent SR appear as three compartments in a row and hence are called “triads.” In cardiac muscle, t-tubules are adjacent to only one SR compartment at a time and hence these are called “diads.”

Triads and diads are critical for excitation-contraction coupling in muscle contraction. In skeletal muscle, PM depolarization causes the voltage sensing L-type calcium channel (LTCC) to undergo a conformational change. Through direct physical coupling, the LTCCs trigger the opening of calcium-activated calcium channels called ryanodine receptors (RyRs) on the SR surface. RyRs release calcium from SR stores and binding of calcium to actin-myosin filaments enables muscle contraction. In skeletal muscle, LTCCs and RyRs are physically coupled and localize to triads. In cardiac muscle, LTCCs and RyRs localize to diads, but LTCCs are not physically coupled to RyRs. Instead, entry of extracellular calcium through LTCCs activates and opens RyRs, which is called calcium-induced calcium release.

In the late 1990s, the molecular basis of triad and diad formation was unclear. Knocking out the LTCC and RyRs in mouse skeletal muscle reduced the number of triad junctions, but the structure of formed triads was morphologically normal (Franzini-Armstrong et al., 1991; Ikemoto et al., 1997). The SR transmembrane proteins triadin and junctin localize to triads, but hydropathy and topology analysis placed the bulk of both proteins in the SR lumen, making it unlikely that the small cytoplasmic portions could directly interact with the PM (Knudson et al., 1993; Jones et al., 1995). These observations suggested that other, yet undiscovered, molecules were the primary structural component of triad junctions.

To identify proteins involved in triad formation, Takeshima et al. (1998) generated monoclonal antibodies against isolated SR vesicles enriched for junctional membranes from rabbit skeletal muscle. Using these antibodies for immunostaining rabbit skeletal muscle cryosections, they identified an antibody that labeled transverse rows corresponding to the location of triad junctions. Further screening of a protein-expression library using this antibody revealed a novel protein, named junctophilin 1 (JPH1) (Takeshima et al., 2000). Through cross-hybridization with mouse cDNA libraries, two other junctophilins were identified which were named junctophilin 2 (JPH2) and junctophilin 3 (JPH3). A fourth junctophilin, junctophilin 4 (JPH4), was later identified by sequence homology (Nishi et al., 2003). Importantly, electron microscopy studies with immunogold labeling confirmed that JPH1 localizes to JMCs in rabbit skeletal muscle (Takeshima et al., 2000).

### Junctophilin Isotypes Are Differentially Expressed in Excitable Tissues

mRNA and protein analyses in mouse and human tissue samples revealed that the four mammalian junctophilins have different expression patterns in excitable tissues. Skeletal muscle expresses both JPH1 and JPH2 at similar levels, while JPH2 is the primary isotype in heart and smooth muscle (Nishi et al., 2000; Takeshima et al., 2000; Ito et al., 2001; Minamisawa et al., 2004; Pritchard et al., 2019; Saeki et al., 2019). JPH3 and JPH4 are broadly expressed in neurons of the brain and nervous system (Takeshima et al., 2000; Nishi et al., 2003, 2000). In addition, JPH3 is expressed in pancreatic beta cells (Li et al., 2016) and JPH4 is expressed in T-cells (Woo et al., 2016), both of which are excitable cell types.

### Junctophilin Domain Structure Facilitates Simultaneous ER and PM Binding

**FIGURE 1:**
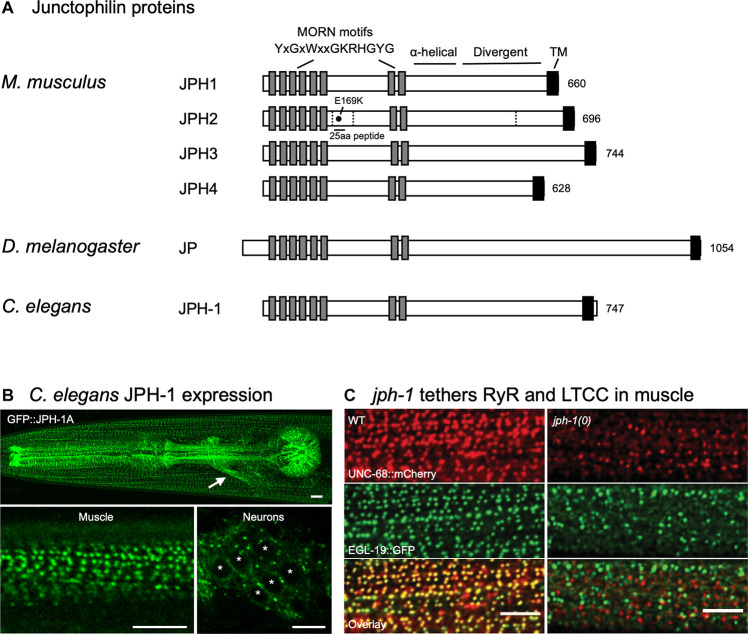
Domain structures of junctophilin proteins and *C. elegans* junctophilin localization and function. **(A)** Domain structures of the four *Mus musculus* (mouse) junctophilins and sole members in *Drosophila melanogaster* (fly) and *Caenorhabditis elegans* (worm). Dashed lines indicate JPH2 cleavage sites. E169K mutation blocks binding to RyR and a 25 aa peptide flanking E169 prevents spontaneous RyR channel opening. Gene accession numbers are: *M. musculus* JPH1 (NP_065629.1), *M. musculus* JPH2 (NP_001192005.1), *M. musculus* JPH3 (NP_065630.1), *M. musculus* JPH4 (NP_796023.2), *D. melanogaster* Jp (NP_523525.2), and *C. elegans* JPH-1 (NP_492193.2). **(B)** Expression of GFP-tagged JPH-1 shows localization to JMCs in *C. elegans* muscles and neurons. Top: Head of a *C. elegans* animal showing GFP::JPH-1 in the pharyngeal muscle and body wall muscle. The white arrow indicates GFP::JPH-1 expression in a bundle of neuronal processes known as the nerve ring. Bottom left: In body wall muscle, JPH-1 localizes to rows of puncta, each one a JMC. Bottom right: In neurons, JPH-1 labels JMCs that form at the periphery of the soma. White asterisks mark neuronal nuclei. **(C)** *jph-1* is required for the co-localization of UNC-68/RyR and EGL-19/LTCC in body wall muscle. *jph-1(0)* is a complete knockout. Scale bars, 5 μm. Panels **(B,C)** reproduced from Piggott et al. (2021).

Junctophilins are conserved from *C. elegans* to humans (Figure 1A). Their domain structure supports their role as an ER–PM contact site protein (Garbino et al., 2009). All junctophilins have eight N-terminal MORN (Membrane Occupation and Recognition Nexus) motifs, which are 14 amino acid motifs with the consensus sequence YxGxWxxGKRHGYG (Figure 1A) (Takeshima et al., 2000; Garbino et al., 2009). Expression studies in amphibian embryos using full-length and truncated rabbit JPH1 showed that MORN motifs are required for targeting JPH1 to the PM (Takeshima et al., 2000). Lipid binding assays showed that recombinant versions of JPH1 and JPH2 that lack the transmembrane domain can bind directly to phospholipids, particularly types enriched in the PM, suggesting that junctophilins can bind to the PM through their MORN motifs (Kakizawa et al., 2008; Bennett et al., 2013).

Following the MORN motifs is a predicted α-helical domain of approximately 70 amino acids that would provide a flexible linker 10.5 nm long, enough to span the 10–12 nm JMCs (Garbino et al., 2009). The divergent region (Figure 1A) has high conservation when comparing the same isotype in different species – for example, >80% sequence identity when comparing human, mouse, and rat isotypes – but low conservation between JPH isotypes, with <20% sequence identity across isotypes in human, mouse, and rat (Garbino et al., 2009). The divergent region might be important for the isotype-specific functions of junctophilin, such as tissue-specific binding sites (Garbino et al., 2009).

A hydrophobic transmembrane domain resides at the C-terminus of junctophilin and anchors junctophilin in the ER/SR membrane (Takeshima et al., 2000). Collectively, these domains allow junctophilins to simultaneously bind both PM and ER membranes and localize to ER–PM JMCs.

### Junctophilins Tether ER and PM Membranes

Junctophilins localize to ER–PM membrane contact sites by simultaneously binding both the ER and PM, thereby functioning as membrane tethers. Expression of JPH1 in amphibian embryos generated ER–PM contacts as well as unnatural ER stacks visible by electron microscopy, demonstrating that junctophilins are capable of tethering membranes (Takeshima et al., 2000). JPH2 knockout is embryonic lethal due to cardiac failure (Takeshima et al., 2000). Embryonic cardiomyocytes from JPH2 knockout mice had fewer 12 nm JMCs, suggesting that JPH2 is required for ER–PM coupling (Takeshima et al., 2000). Using an inducible heart-specific JPH2 knockdown to bypass embryonic lethality, van Oort et al. (2011) showed that JPH2 knockdown reduces ER–PM contact site number. Conversely, heart-specific overexpression of JPH2 in mouse increased ER–PM contact site area and also generated convoluted membrane structures visible by electron microscopy (Guo et al., 2014), reminiscent of the ER stacks generated by JPH1 overexpression in amphibian embryos (Takeshima et al., 2000). Taken together, these early studies provide strong evidence that junctophilin acts as an ER–PM tether.

## Functional Studies of Mammalian JPH1 and JPH2 in Muscles

Over the past two decades, extensive studies using knockdown and knockout mice and relevant cell lines have supported critical roles of junctophilins in various types of muscle. Below, we will review functional studies of each junctophilin (summarized in Table 1).

**TABLE 1:** Functions of junctophilins from invertebrates to mammals.

Organism	Gene name	Tissue distribution	Deficiency phenotypes	References*
Mouse	JPH1	Skeletal muscle	Perinatal lethality	Ito et al., 2001
			Deformed triads	Golini et al., 2011
			Impaired excitation-contraction coupling	Hirata et al., 2006
			Reduced LTCC and RyR co-localization	
			Reduced store-operated calcium entry	
Mouse	JPH2	Skeletal and heart muscle	Embryonic lethality	Takeshima et al., 2000
			Fewer ER–PM contacts	Hirata et al., 2006
			Deformed triads	Reynolds et al., 2013
			Reduced store-operated calcium entry	Golini et al., 2011
			Disrupted t-tubules	
			Reduced LTCC and RyR co-localization	van Oort et al., 2011
			Impaired excitation-contraction coupling	Wang et al., 2014
			Spontaneous calcium sparks	Pritchard et al., 2019
			Increased smooth muscle contraction	
Mouse	JPH3 and JPH4	Brain	Motor discoordination	Kakizawa et al., 2007
			Impaired memory	Moriguchi et al., 2006
			Abolished afterhyperpolarization	Sahu et al., 2019
			Reduced LTCC, RyR, SK channel co-localization	
*D. melanogaster*	Jp	Muscles and neurons	Reduced lifespan	Calpena et al., 2018
			Impaired flight	
			Deformed muscle ultrastructure	
			Cardiac dysfunction	
			Neurodegeneration	
*C. elegans*	*jph-1*	Muscles and neurons	Stunted growth	Piggott et al., 2021
			Motor discoordination	
			Reduced LTCC and RyR co-localization	
			Reduced axon regeneration	
			Impaired synaptic transmission	

*Only select references, due to space limitations.

### JPH1 and JPH2 Form and Stabilize Triad Junctions and T-Tubules in Muscles

JPH1 was first identified as a protein that localizes to triad junctions in rabbit skeletal muscle (Takeshima et al., 2000). Skeletal muscle from newborn JPH1 knockout mice has abnormal SR morphology and fewer triads, consistent with a role for JPH1 in triad formation (Ito et al., 2001). JPH1 knockout mice also have weaker muscle contraction and die within a day of birth due to suckling defects, suggesting that JPH1-mediated triad assembly is critical for muscle function (Ito et al., 2001). In adult mice, dual knockdown of JPH1 and JPH2 disrupts existing triads (Hirata et al., 2006). Therefore, JPH1 and JPH2 are required for both triad development and stabilization. Junctophilin’s ability to directly bind membranes likely contributes to this role. However, interactions with JMC-localized proteins such as the LTCC and RyRs, which will be discussed later in this article, may contribute to both membrane tethering and targeting JPH1 and JPH2 to triads.

Extensive studies have shown that JPH2 is also involved in the development and stabilization of t-tubules. The arrival of JPH2 at the rat cardiomyocyte PM coincides with the start of membrane invagination at P10 (Ziman et al., 2010). Constitutive heart-specific knockdown of JPH2 impedes t-tubule development (Chen et al., 2013; Reynolds et al., 2013). Knockdown of JPH2 in rat myocytes disrupts the organization of existing t-tubules (Wei et al., 2010). In an inducible mouse heart failure model, where JPH2 undergoes proteolytic cleavage, t-tubules are disrupted, suggesting that intact JPH2 is required for t-tubule maintenance (Wu et al., 2014). JPH2 overexpression has the opposite effect on t-tubule organization. Heart-specific JPH2 overexpression in mouse accelerates t-tubule development (Reynolds et al., 2013). In trans-aortic banded mice, which normally exhibit t-tubule disruption, JPH2 overexpression protects t-tubule organization (Guo et al., 2014). Given their ability to simultaneously bind t-tubule and SR membranes, it is conceivable that JPH2 generates and maintains t-tubule organization by tethering t-tubules to the SR.

These findings highlight junctophilin’s role as a structural protein that shapes the muscle ultrastructure. However, as we will discuss next, junctophilins also play a key role in positioning and regulating ion channels to facilitate excitation-contraction coupling.

### JPH1 and JPH2 Facilitate Localization and Coupling of Calcium Channels for Excitation-Contraction Coupling

The co-localization of ER- and PM-localized calcium channels at JMCs is essential for effective excitation-contraction coupling. In skeletal muscle, depolarization of the PM causes LTCCs to trigger the opening of physically linked RyRs and release calcium from SR stores. In heart muscle, PM depolarization opens LTCCs and the resulting calcium influx triggers the opening of nearby RyRs. In both cases, coupling of PM-localized LTCCs and SR-localized RyRs is crucial for effective conversion of PM depolarization to SR calcium release which drives muscle contraction.

Pioneering studies showed that embryonic cardiomyocytes isolated from JPH2 knockout mice exhibited random, unsynchronized calcium transients (Takeshima et al., 2000). Calcium transients occurred even when extracellular calcium was removed, indicating that JPH2 is required for coupling SR calcium release to extracellular calcium entry (Takeshima et al., 2000). In cardiomyocytes and intact hearts from JPH2 knockdown mice, electrical stimulation induced calcium transients that were smaller and irregular compared to wild-type (Chen et al., 2013; Reynolds et al., 2013). This occurred with no change to LTCC and RyR protein levels, suggesting that LTCCs and and/or RyRs were mis-localized or mis-regulated when JPH2 was depleted.

Multiple studies have demonstrated that junctophilins interact with both RyRs and the LTCC. Super-resolution microscopy of rat cardiomyocytes showed that roughly 80% of RyRs and JPH2 co-localize (Jayasinghe et al., 2012). Co-immunoprecipitation studies in rabbit skeletal muscle, mouse heart muscle, and transiently transfected HEK293 cells demonstrated binding between JPH1 or JPH2 and RyRs (Phimister et al., 2007; Golini et al., 2011; van Oort et al., 2011; Beavers et al., 2013). Similarly, in rabbit skeletal muscle binding was seen between JPH1 or JPH2 and the LTCC (Golini et al., 2011). Moreover, cardiomyocytes isolated from heart-specific JPH2 knockdown mice had reduced LTCC and RyR co-localization (van Oort et al., 2011). These cardiomyocytes had normal depolarization-stimulated calcium influx through LTCCs but smaller cytosolic transients. These findings suggest that JPH2 is required for co-localizing LTCCs and RyRs so that extracellular calcium entry through LTCCs can efficiently stimulate RyR-mediated SR calcium release.

Another elegant study addressed the role of direct protein interactions in localizing LTCCs (Nakada et al., 2018). In GLT myotubes, expression of LTCCs with a point mutation that blocked binding to JPH1 and JPH2 caused LTCCs to become diffuse and no longer co-localize with RyR. Fewer myotubes produced calcium transients in response to electric field stimulation, and those that produced transients had smaller amplitudes. Moreover, mis-localizing LTCCs by introducing a truncated JPH1 lacking the transmembrane domain that itself was mis-localized resulted in reduced calcium amplitude and reduced contraction strength in mouse skeletal muscle. Therefore, JPH2 likely mediates excitation-contraction coupling by binding to and localizing LTCCs near RyRs.

Altogether, this substantial body of work, performed in multiple systems, shows that JPH1 and JPH2 directly bind the LTCC and RyRs to facilitate their co-localization and enable efficient excitation-contraction coupling.

### JPH2 Controls Gating of Calcium Channels

A growing body of evidence suggests that junctophilins not only couple ER- and PM-localized calcium channels but also play a role in channel gating. Cardiomyocytes have spontaneous local increases in cytosolic calcium called “calcium sparks” that are caused by opening of RyRs (Cheng et al., 1993). Cardiomyocytes from inducible JPH2 knockdown mouse hearts have larger and more frequent spontaneous calcium sparks with no change in RyR expression level, suggesting that JPH2 is required for keeping RyRs closed (van Oort et al., 2011; Wang et al., 2014). Cardiomyocytes from mice expressing JPH2 with the E169K mutation, a residue required for RyR binding, also have more frequent spontaneous calcium sparks, suggesting that JPH2 directly binds and gates RyRs (Figure 1A) (Beavers et al., 2013). Supporting this, addition of a 25aa peptide flanking E169 to permeabilized cardiomyocytes from JPH2 knockdown mice abolishes spontaneous calcium release (Beavers et al., 2013).

Further evidence that JPH2-binding controls RyR gating comes from *in vitro* single-channel recordings using microsomes extracted from mouse hearts and reconstituted in planar lipid bilayers. RyRs from inducible JPH2 knockdown mouse hearts have higher open probability than RyRs from wild-type mice, suggesting that JPH2 prevents RyR opening (Wang et al., 2014). The 25aa JPH2-derived peptide flanking E169 reduces RyR opening probability to wild-type levels (Beavers et al., 2013). Conversely, JPH2 knockdown reduced spontaneous calcium spark frequency in HL-1 immortalized cardiomyocytes (Landstrom et al., 2011). This response, which was the opposite of results obtained from cardiomyocytes from JPH2 knockdown mice, may have been different due to the immortalized nature and altered calcium handling of HL-1 cells.

JPH1 and JPH2 dual knockdown in C2C12 myotubes *in vitro* has been shown to impair calcium influx through LTCC (Nakada et al., 2018). These findings were seen with unchanged membrane expression of LTCC and suggest that junctophilins may gate LTCC, though could also be explained by attenuation of a retrograde signal from RyR (Nakai et al., 1996).

### JPH1 and JPH2 Are Required for Store-Operated Calcium Entry in Skeletal Muscle

When ER calcium stores are depleted, they can be replenished through a process called store-operated calcium entry (SOCE). ER calcium sensor STIM1 senses the drop in calcium concentration and relocalizes to ER–PM contacts, where it activates PM-localized calcium channel Orai1 to allow the entry of extracellular calcium. Sarco/ER calcium transport ATPase (SERCA) pumps then take up the calcium into the ER (Serwach and Gruszczynska-biegala, 2020).

*In vitro* and *in vivo* studies in skeletal muscle have demonstrated roles for junctophilins in SOCE. Knockdown of both JPH1 and JPH2 in cultured myotubes reduced pharmacologically-induced SOCE, measured by the quenching of intracellular Fura-2 by the entry of extracellular Mn^2+^ through SOCE channels (Hirata et al., 2006; Li et al., 2010). Adenovirus-mediated knockdown of both JPH1 and JPH2 in mouse skeletal muscle also reduced SOCE, indicated by the reduction of calcium in t-tubules (Hirata et al., 2006). This was associated with a slower recovery of voltage-induced calcium release after SR calcium depletion, consistent with impaired SOCE (Hirata et al., 2006). Skeletal muscle from these mice was found to have deformed triads (Hirata et al., 2006). As STIM1 and Orai1 interact at ER–PM contacts, an appealing hypothesis is that JPH1 and JPH2 are required for SOCE in skeletal muscle because STIM1-Orai1 interactions occur at JMCs generated by junctophilins.

### JPH2 Couples RyRs and BK Channels in Smooth Muscle

Like cardiac and skeletal muscle, smooth muscle contraction is primarily regulated by an increase in cytosolic calcium concentration (Allen and Walsh, 1994). However, the regulation of cytosolic calcium in smooth muscle is a complex process that can be modulated by various signaling pathways (Kuo and Ehrlich, 2015). Similar to cardiac muscle, membrane depolarization triggers calcium-induced calcium release involving the LTCC and RyRs (Sanders, 1985). Alternatively, extracellular ligands bind to PM-localized receptors which generate 1,4,5-trisphosphate (IP3). IP3 diffuses across the cytosol and activates SR-localized calcium channels known as IP3 receptors, which release SR calcium (Sanders, 1985). Both calcium-induced and IP3-induced calcium release would appear to benefit from close coupling of the ER and PM. Indeed, LTCCs and RyRs co-localize at JMCs in smooth muscle (Moore et al., 2004). However, LTCCs and RyRs in smooth muscle exhibit “loose” coupling, wherein LTCC opening does not necessarily trigger RyR activation, and RyRs can open spontaneously to generate calcium sparks (Collier et al., 2000). Furthermore, unlike cardiac and skeletal muscle, smooth muscle lacks t-tubules to enhance ER–PM contact area. Therefore, JMCs, and by extension, junctophilins, may not be as critical for excitation-contraction coupling in smooth muscle as in cardiac or skeletal muscle.

Studies on junctophilins in smooth muscle have focused on coupling between RyRs and large conductance calcium-activated potassium channels, commonly called BK channels (short for “big potassium”). Calcium sparks from spontaneous opening of RyRs activate nearby BK channels and generate an outward potassium current (Nelson et al., 1995). This hyperpolarizes the PM, suppressing depolarization-dependent calcium influx and relaxing the muscle (Bolton and Imaizumi, 1996). JPH2 is the most abundant junctophilin isotype in vascular smooth muscle (Pritchard et al., 2019; Saeki et al., 2019). JPH2 localization overlaps with RyR and BK channels in vascular smooth muscle, where it directly binds BK, suggesting JPH2 couples RyRs and BK channels (Pritchard et al., 2019; Saeki et al., 2019). Calcium sparks, visualized using TIRF microscopy and the fluorescent calcium indicator Fluo-4 AM, occur near JPH2 clusters (Saeki et al., 2019). The outward current activated by spontaneous calcium sparks is reduced when JPH2 is knocked down, consistent with a role for JPH2 in coupling RyRs and BK channels (Pritchard et al., 2019; Saeki et al., 2019). Further supporting this, JPH2 knockdown causes increased vascular smooth muscle contraction (Pritchard et al., 2019; Saeki et al., 2019). Therefore, JPH2 plays an important role in maintaining vascular smooth muscle resting tone by coupling RyRs to BK channels. As JPH2 expression was detected in mouse stomach and lung (Takeshima et al., 2000), which contain smooth muscle, further study will be required to determine if this function of JPH2 is conserved across smooth muscle in different tissues.

### Heart Stress Induces Cleavage of JPH2 to Regulate Transcription in Heart Muscle

Excessive muscle contraction and heart stress can cause prolonged elevation of cytosolic calcium (Gissel, 2000; Stary and Hogan, 2000; Dhalla et al., 2008). Such increases in calcium have been shown to disrupt excitation-contraction coupling in muscle fibers without altering LTCC or RyR levels (Lamb et al., 1995). Evoking high calcium levels was found to cause proteolysis of JPH1 and JPH2 in mouse skeletal muscle, raising the possibility that disrupted excitation-contraction coupling was due to junctophilin degradation (Murphy et al., 2013). In support of this, exposure of rat muscle fiber to high calcium degraded JPH1 and reduced the muscle’s contractile force (Murphy et al., 2013).

Junctophilin cleavage was found to be dependent on the calcium-activated protease calpain, as administering a calpain inhibitor to an inducible mouse model of heart failure blocked JPH2 cleavage and the associated t-tubule disruption and abnormal calcium handling (Wu et al., 2014). Subsequent studies revealed three putative calpain cleavage sites in JPH2 (Figure 1A) (Guo et al., 2015). Moreover, an N-terminal cleavage product of JPH2 (JPH2-NT) produced by heart stress is directed to the nucleus where it directly binds DNA (Guo et al., 2018). Microarray analysis of cultured cardiomyocytes overexpressing JPH2-NT demonstrated that JPH2-NT could alter transcription, likely by competing with transcription factors for DNA binding. Guo et al. further showed that JPH2-NT overexpression protected mouse hearts against stress, and in the converse experiment, preventing nuclear accumulation (by deleting the NLS in endogenous JPH2) exacerbated symptoms of heart stress.

Disrupted excitation-contraction coupling is considered to be a common step in the progression of heart failure (Ibrahim et al., 2011). It can be argued that reducing excitation-contraction coupling by cleaving JPH2 provides a temporary solution to excessive cardiac muscle contraction, which can lead to hypertrophy and eventually heart failure (Hunter and Chien, 1999). The cleavage product JPH2-NT has an additional protective role in inducing transcriptional reprogramming and attenuating the progression of heart failure (Guo et al., 2018). Terminating heart failure-inducing signals in a mouse model results in normalization of JPH2 levels, improvements in excitation-contraction coupling, and reversal of heart failure (Wu et al., 2014). Therefore, while JPH2 cleavage may be harmful under conditions of prolonged heart stress, it may be a beneficial response to short-term stress. Additional studies in other models will be required to test this model.

## Functional Studies of Mammalian JPH3 and JPH4 in Neurons

### Neuronal Junctophilins Are Involved in Motor Coordination and Learning

JPH3 and JPH4 are broadly expressed in neurons of the brain and nervous system (Takeshima et al., 2000; Nishi et al., 2003, 2000). A growing body of evidence suggests that JPH3 and JPH4 have overlapping roles mediating learning and motor control through the regulation of intracellular calcium signaling in neurons. JPH3 or JPH4 knockout mice show slight impairment of motor coordination, which progresses with aging in the case of JPH3 knockout (Nishi et al., 2002; Kakizawa et al., 2007; Seixas et al., 2012). In contrast, JPH3 and JPH4 double knockout (JPH DKO) mice die 3-4 weeks after birth, though this can be prevented by switching their food from dry pellets to a wet paste, suggesting lethality is due to defects in the circuitry controlling saliva secretion (Moriguchi et al., 2006). JPH DKO causes severe defects in motor coordination, learning, and memory, suggesting that JPH3 and JPH4 have important overlapping roles in the brain (Moriguchi et al., 2006; Kakizawa et al., 2007).

### JPH3 and JPH4 Are Required for Neuronal Afterhyperpolarization Currents

The molecular basis for the neurological defects in JPH DKO may lie in the production of afterhyperpolarization (AHP) currents. The depolarization and repolarization phases of an action potential are followed by AHP, where the neuron’s membrane potential falls below the normal resting potential, and is one factor determining action potential frequency (Andrade et al., 2012). Slice recordings in hippocampal CA1 neurons obtained from JPH DKO mice showed that AHP is absent (Moriguchi et al., 2006). Inhibitors of NMDA receptor cation channels, RyR channels, and small conductance calcium-activated potassium (SK) channels abolished AHP in wild-type neurons but had no additional effect on currents in JPH DKO neurons, leading the authors to propose that neuronal junctophilins are required for the production of AHP currents by coupling NMDA receptors, RyRs, and SK channels (Moriguchi et al., 2006). Slice recordings in cerebellar Purkinje cells obtained from JPH DKO mice or treated with channel inhibitors showed similar effects on AHP (Kakizawa et al., 2007). Moreover, addition of the SK channel enhancer EBIO restored AHP in Purkinje cells from JPH DKO animals, suggesting that SK channels are functional in JPH DKO but require junctophilin-mediated coupling to RyR for activation (Kakizawa et al., 2007).

Unlike in muscle, neuronal junctophilin knockout caused no disruption to ER–PM membrane contact sites detectable by electron microscopy (Nishi et al., 2002; Moriguchi et al., 2006; Kakizawa et al., 2007). Therefore, it appears that junctophilin’s main role in neurons is not to tether membranes but facilitate channel localization. Indeed, in cultured hippocampal CA1 neurons, studies using super-resolution microscopy showed that JPH3 and JPH4 are required to maintain the co-assembly of LTCCs, RyRs, and SK channels, and that disruption of this co-assembly leads to impaired AHP and more frequent action potentials (Sahu et al., 2019). These results suggest a model where junctophilins couple PM-localized cation channels (e.g., NMDA receptor, CaV1.3), ER-localized RyRs, and PM-localized SK channels for intracellular communication to link membrane depolarization to AHP current generation and ultimately control action potential frequency.

It is currently unclear how altered AHP might manifest as motor coordination and learning defects. Recordings from Purkinje cell and hippocampal CA1 neurons have shown that JPH DKO causes defects in long term potentiaton and long term depression (Moriguchi et al., 2006; Kakizawa et al., 2007). Future work will be required to determine the mechanism causing behavioral defects in JPH DKO animals.

### A Trinucleotide Repeat Expansion in *JPH3* Causes Huntington’s Disease-Like 2

Shortly after the discovery of junctophilins, a CAG/CTG repeat expansion in an alternatively spliced exon of *JPH3* was found to cause Huntington disease-like 2 (HDL2), a disease clinically indistinguishable from Huntington’s disease (Holmes et al., 2001). Based on studies in cultured cells and transgenic mice, three disease mechanisms have been proposed: (1) sequestration of *JPH3* mRNA carrying the expanded repeat and subsequent loss of function (Seixas et al., 2012), (2) toxic gain of function by *JPH3* mRNA carrying the expanded repeat (Rudnicki et al., 2007), and (3) toxic gain of function peptides translated from either the sense or antisense strand carrying the expanded repeat (Wilburn et al., 2011). Additional studies will be required to determine if HDL2 pathogenesis involves the loss of endogenous JPH3 function, is caused by CUG repeats which merely happen to be located at the *JPH3* locus, or a combination of both.

## Function and Mechanism Conservation: Insights From Invertebrates

Recent studies from the invertebrates *C. elegans* and *D. melanogaster* have demonstrated that the functions and mechanisms of junctophilins are highly conserved. Invertebrates have only one junctophilin, facilitating the study of junctophilin without concerns of redundancy (Garbino et al., 2009) (Figure 1A).

In *D. melanogaster*, knockdown or overexpression of the sole junctophilin caused structural defects in skeletal and cardiac muscle structure which were accompanied by functional deficits (Calpena et al., 2018). However, the localization of fly junctophilin is not yet reported; therefore, its relationship to ER–PM calcium channels remains unaddressed. Interestingly, junctophilin overexpression appears to be protective against neuronal degeneration caused by expression of human huntingtin exon 1 carrying expanded polyglutamine repeats (Calpena et al., 2018). Genetic interaction experiments suggest that this role of junctophilin may involve the Notch signaling pathway.

In *C. elegans*, expression of GFP-tagged JPH-1 under the control of the *jph-1* promoter showed that *jph-1* is expressed in all muscles, and likely most neurons (Figure 1B) (Piggott et al., 2021). In muscles, JPH-1 localizes to longitudinal rows of puncta that match the pattern of repeating sarcomere units. These JPH-1 puncta co-localize with both the ER-localized UNC-68/RyR and PM-localized LTCC subunit EGL-19, implying that JPH-1 localizes to ER–PM contact sites. In neurons, JPH-1 co-localizes with the ER–PM contact site protein Extended-SYnaptoTagmin 2 (ESYT-2). Together, these data demonstrate that *C. elegans* junctophilin is expressed in excitable tissues and localizes to ER–PM contact sites, a feature that is conserved from *C. elegans* to mammals.

*jph-1* knockout animals are viable, allowing the study of junctophilin function in whole animals (Piggott et al., 2021). *jph-1* knockout animals show stunted growth and slow and uncoordinated movement. The stunted growth is likely due to reduced nutrient intake caused by weak contraction of the pharyngeal muscle, the organ that draws in and crushes bacteria for eating, as expression of JPH-1 in the pharyngeal muscle restored both muscle contraction and animal growth. The impaired movement is likely due to defective body wall muscle contraction, as expression of JPH-1 in body wall muscle rescued movement. Consistent with a role of *jph-1* in coupling ER- and PM-localized calcium channels, *jph-1* knockout abolishes co-localization between LTCC subunit EGL-19 and UNC-68/RyR in muscles (Figure 1C). Interestingly, precise subcellular localization of JPH-1 in both muscle and neurons depends on *unc-68*/RyR. It was reported that in rat cardiomyocytes, RyR localization to muscle triads precedes JPH2 arrival, suggesting that the targeting of junctophilins by RyRs may be conserved (Ziman et al., 2010). Mammalian JPH1 and JPH2 directly bind to RyR (Phimister et al., 2007; van Oort et al., 2011). Thus, it is possible that junctophilin targeting may involve directly binding to RyR already localized at MCSs.

Studies from *C. elegans* have also opened new directions for junctophilin research, particularly in neurons. *jph-1* knockout animals were found to have reduced axon regrowth after injury (Piggott et al., 2021). Regrowth was rescued by expression of JPH-1 in pharyngeal muscle, raising the intriguing possibility that gut nutrients may impact neuronal injury response. In cholinergic neurons, JPH-1 surrounds synaptic release sites labeled by the vesicular acetylcholine transporter UNC-17. Pharmacological assays showed that *jph-1* is required for synaptic transmission at the neuromuscular junction, a previously undescribed role for junctophilin. The ER–PM contact site protein ESYT-2 was also found to be required for synaptic transmission, echoing findings from *D. melanogaster* (Kikuma et al., 2017). Unexpectedly, mutating both *jph-1* and *esyt-2* restored wild-type synaptic transmission, in a display of mutual suppression (Piggott et al., 2021). While the underlying mechanism remains to be addressed, *jph-1* and *esyt-2* appear to have antagonistic roles in neuromuscular synaptic transmission. These observations hint at a delicate balance of different classes of tethering molecules at ER–PM junctions.

Gene duplication events provide the opportunity for isotypes to evolve specialized roles (Ohno, 1970). As vertebrates have four junctophilins and invertebrates have one, the ancestral function of junctophilin is likely closer to invertebrate junctophilins. Studies in *C. elegans* have shown that junctophilins likely have a conserved role in coupling ER- and PM-localized calcium channels, suggesting this is an ancestral role. Although a role in ER–PM tethering has yet to be experimentally demonstrated in fly or *C. elegans*, the high conservation of the overall domain structure and MORN motifs across species suggests that ER–PM tethering may also be an ancestral role.

## Concluding Remarks

Since the discovery of junctophilins two decades ago, great progress has been made toward revealing their cell biology and functional importance. Compared to membrane contact sites, junctophilins are unique in that each isotype displays critical roles in a cell-type specific manner. JPH1 and JPH2 are expressed in muscle and contribute to muscle structure by generating JMCs, triads/diads, and t-tubules. JPH1 and JPH2 bind to LTCCs and RyRs to facilitate their co-localization and enable efficient excitation-contraction coupling. In addition to these well-established roles, there is evidence that JPH1 and JPH2 regulate SOCE in skeletal muscle, JPH2 controls gating of RyRs and acts as a transcriptional regulator in heart muscle, and JPH2 couples BK and RyR channels in smooth muscle. JPH3 and JPH4 are required in neurons for the generation of AHP currents and have roles in motor coordination and learning.

Emerging findings have begun to show additional roles for JPH3 and JPH4 in other cell types. JPH3 is required for glucose-stimulated insulin release in pancreatic beta cells (Li et al., 2016). JPH4 is required for SOCE in T-cells and dorsal root ganglia, showing that a role in SOCE is conserved in at least three out of four junctophilin isotypes (Woo et al., 2016; Hogea et al., 2021). JPH4 was found to facilitate SOCE by recruiting STIM1 and Orai1 to ER–PM contacts, providing a possible mechanism for how JPH1 and JPH2 might regulate SOCE in skeletal muscle (Woo et al., 2016; Hogea et al., 2021). *In vitro* studies demonstrated that JPH3 and JPH4 interact with Cav2.1 P/Q-type calcium channels and Cav2.2 N-type calcium channels and modify their inactivation rates (Perni and Beam, 2021). This study also found that JPH3 and JPH4 differentially interact with RyR isotypes, which may explain why the brain expresses two different junctophilin isotypes.

The roles of junctophilin in neurons are among the biggest open questions that remain to be addressed. More specifically: (1) Does impaired AHP, whichis observed in neurons obtained from JP3 and JPH4 knockout mice, cause the motor coordination and learning defects found in JPH3 and JPH4 knockout animals? And if so, what is the mechanism? (2) While JPH3 and JPH4 are broadly expressed in the nervous system, they have only been studied in cerebellar Purkinje cells, hippocampal CA1 neurons, and certain sensory neurons. Given that different neurons have different ion channel expression patterns, what are the functions of JPH3 and JPH4 in unexamined neurons? (3) Studies in *C. elegans* demonstrated that junctophilin and extended-synaptotagmin have antagonistic roles in synaptic transmission. The mechanism behind their roles in synaptic transmission and genetic interaction remain to be investigated. (4) What is the disease mechanism responsible for HDL2 in patients with *JPH3* trinucleotide repeats? Addressing these questions will not only further our understanding of junctophilins, but also how calcium signaling in neurons can regulate neuronal function.

## Author Contributions

CP wrote the original draft, along with inputs and edits from YJ. Both authors contributed to the article and approved the submitted version.

## Conflict of Interest

The authors declare that the research was conducted in the absence of any commercial or financial relationships that could be construed as a potential conflict of interest.
